# Comparison of the results of two chest tube managements during an enhanced recovery program after video‐assisted thoracoscopic lobectomy: A randomized trial

**DOI:** 10.1111/1759-7714.13183

**Published:** 2019-09-02

**Authors:** Zihan Cui, Yuejuan Zhang, Chun Xu, Cheng Ding, Jun Chen, Chang Li, Jun Zhao

**Affiliations:** ^1^ Department of Thoracic Surgery The First Affiliated Hospital of Soochow University, Medical college of Soochow University Suzhou China

**Keywords:** Chest tube, ERAS, non‐small cell lung cancer, video‐assisted thoracoscope lobectomy

## Abstract

**Background:**

This study compared the results of the application of two different chest tube management systems; a drainage ball with low negative pressure and the more commonly used chest tube with water‐sealed bottle, after video‐assisted thoracoscopic (VATS) lobectomy.

**Methods:**

A total of 60 patients undergoing lobectomy were enrolled into this prospective open label randomized clinical trial and equally divided into two groups. The data collected in the trial included age, gender, forced expiratory volume in 1 second (FEV1), blood loss, operation time, drainage volume, drainage time, length of stay, postoperative pain score according to the Visual Analogue Scale (VAS) within 24 hours after surgery and chest tube removal. This study was registered at ClinicalTrials.gov (NCT03598296).

**Results:**

The characteristics of the patients were similar in both groups. Group ball patients had a lower pain score (after operation: 3.47 ± 1.80 vs. 6.20 ± 1.56, *P* < 0.001; after removal of chest tube: 1.47 ± 1.28 vs. 3.00 ± 1.29, *P* < 0.001); less analgesic used (2.83 ± 2.09 times vs. 5.00 ± 3.24 times, *P* = 0.003); less drainage time (upper tube: 3.89 ± 1.63 days vs. 5.10 ± 2.02 days, *P* = 0.048; lower tube: upper lobe 4.84 ± 1.61 days vs. 5.90 ± 1.52 days, *P* = 0.041; lower lobe: 3.82 ± 1.08 days vs. 5.70 ± 2.63 days, *P* = 0.042) and shorter length of stay (5.40 ± 1.65 days vs. 6.37 ± 1.99 days, *P* = 0.045). All other related parameters were similar in both groups.

**Conclusions:**

For patients undergoing lobectomy, using a drainage ball with negative pressure could reduce hospitalization days and postoperative pain compared with the more commonly used chest tube with water‐sealed bottle when a strict postoperative curative procedure was performed.

## Introduction

The concept of Enhanced Recovery after Surgery (ERAS) has been used in elective surgery since the 1990s^1^ and is nowadays well‐accepted by surgeons. An enhanced recovery pathway (ERP) is a multimodal and evidence‐based method which combines varieties of elements aimed at many objects, including enhancing recovery after an operation, reducing complications and shortening hospitalization. ERP has demonstrated evident superiority compared with the more conventional approach, and has been shown to improve therapeutic outcomes in almost all major surgical specialties.^2^, ^3^

Non‐small cell lung cancer (NSCLC) is a common malignancy of the lung in China. Statistics show that NSCLC ranks first in incidence among all malignancies and is the main leading cause of cancer death in China. Anatomic pulmonary resection is a majority component of multimodal therapy according to NCCN guidelines. Currently, video‐assisted thoracoscopic surgery (VATS) is an essential element to ERP of thoracic surgery. Thoracoscopic lobectomy with lymphadenectomy as a minimally invasive procedure has become the standard surgery for invasive NSCLC. However, in lobectomy, there is limited and lack of persuasive evidence to prove that ERP influences a therapeutic outcome. According to a recent systematic review of six studies, only one was a randomized trial.^4^ Well‐designed randomized clinical trials are needed to provide conclusive evidence for the role of the ERAS protocols in VATS lobectomies.^5^

Approximately 20 years ago, Cerfolio *et al*. identified modifiable and nonmodifiable factors in ERAS of pulmonary resections.^6^, ^7^ The most considered modifiable factors have been the management of chest tubes, pain control and social support plans. In order to prevent air leakage or pleural effusion, the classic and widely accepted practice has been to insert two tubes in the apical and basal positions at the end of the operation. However, despite the many benefits, this approach is associated with various complications such as pain, infection and blockage which may prolong the hospitalization of patients. In 2003, Alex *et al*. reported on the first single chest drain after lobectomy in a nonrandomized study. Their study proved that, compared with the conventional two‐drain method, a single chest drain has many benefits such as draining fluid and air effectively, reducing postoperative pain and lowering the cost of treatment.^8^ Since then, four other reports of randomized controlled trials have proven the safety of using a single chest drain. In addition, Inaba *et al*. has compared the efficacy of small versus large chest tubes for use in thoracic trauma but no statistically significant difference was found.^9^ Recently, Ueda and colleagues reported the validity of their original strategy for omitting chest tube drainage after major lung resection.^10^ However, there is no generally accepted approach to chest tube management and clinical experience is still the most important basis in chest tube strategy.^11^

In our institution, we routinely use a drainage ball (Xinda Medical Equipment Company, Suzhou, Jiangsu, China) which has the function of negative pressure suction (maximum pressure about 10 kpa, and the actual value of negative pressure will decrease when the drainage ball is filled) to cure those mild patients. It has achieved some satisfactory outcomes. The purpose of this study was to assess the impact of using a drainage ball with low negative pressure to replace the more commonly used chest tube with a water‐sealed bottle to cure patients undergoing VATS lobectomy.

## Methods

This single central trial was conducted in the thoracic surgery of The First Affiliated Hospital of Soochow University. The study protocol was approved by the Ethics Committee of The First Affiliated Hospital of Soochow University, Suzhou, China, and registered at ClinicalTrials.gov (NCT03598296). All participants gave their written informed consent.

Inclusion criteria were lobectomy by video‐assisted thoracic surgery (VATS), age range from 30 to 70 years and a diagnosis of invasive carcinoma by frozen section. Patients who had a previous history of pulmonary surgery, severe pulmonary or cardiac disease, metastasis and serious pleural adhesions were excluded from the study.

**Figure 1:**
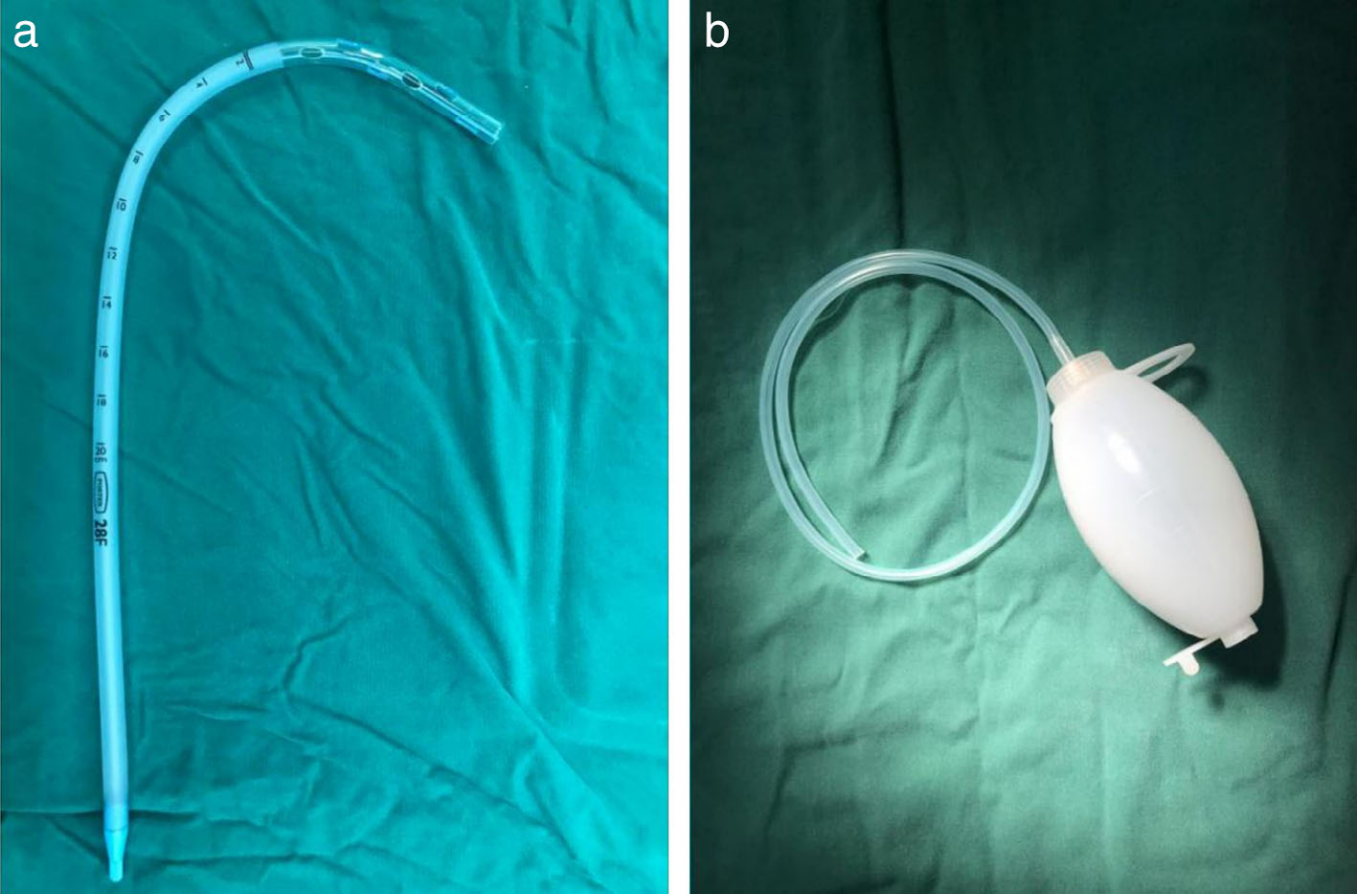
(**a**) Chest tube (28F). (**b**) Drainage ball.

**Figure 2:**
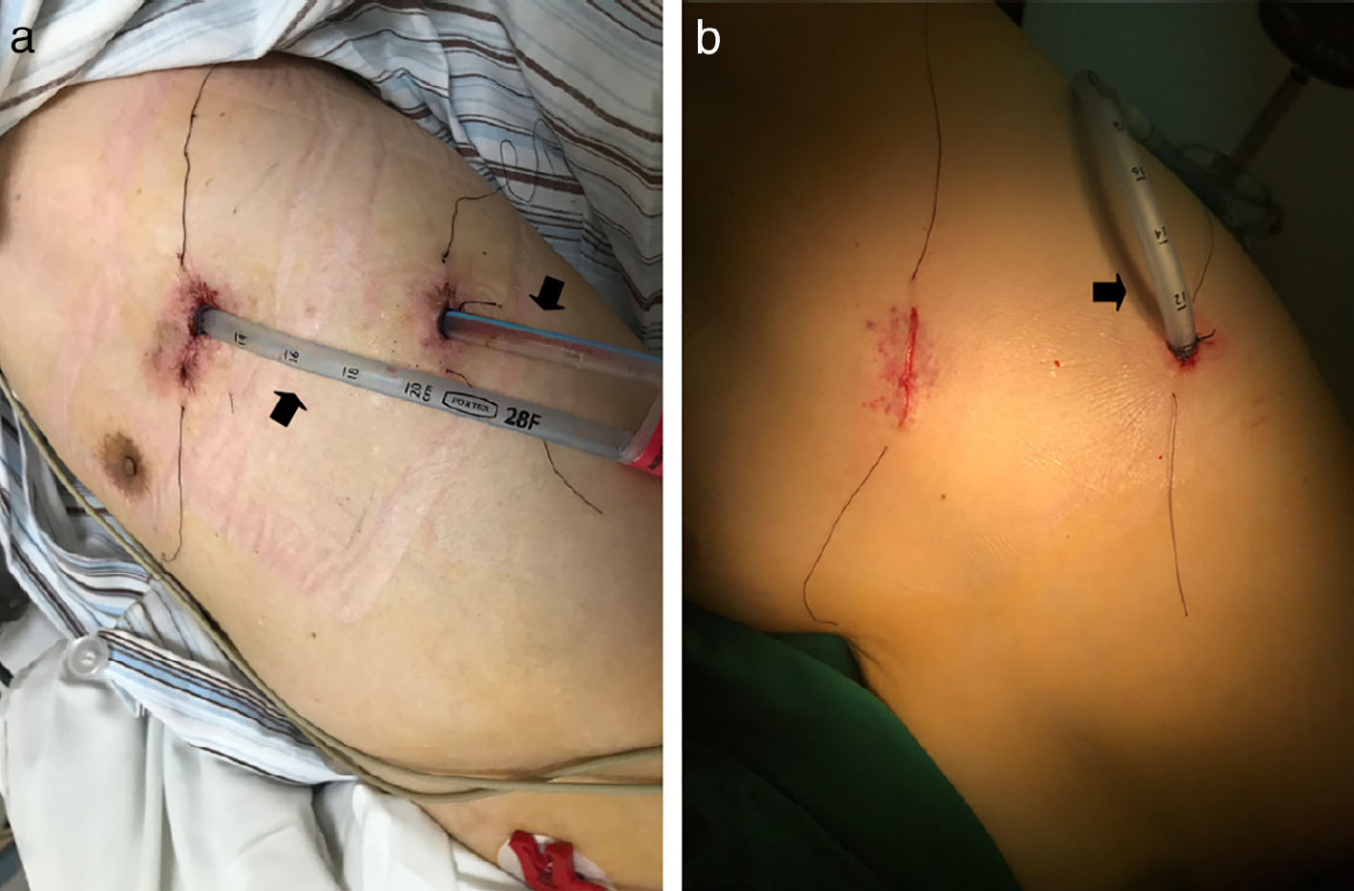
(**a**) Patient accepted left upper lobectomy in Group tube. Two commonly used chest tubes (28F, arrow) were inserted. (**b**) Patient accepted left lower lobectomy in Group tube. One commonly used chest tube (28F, arrow) was inserted.

**Figure 3:**
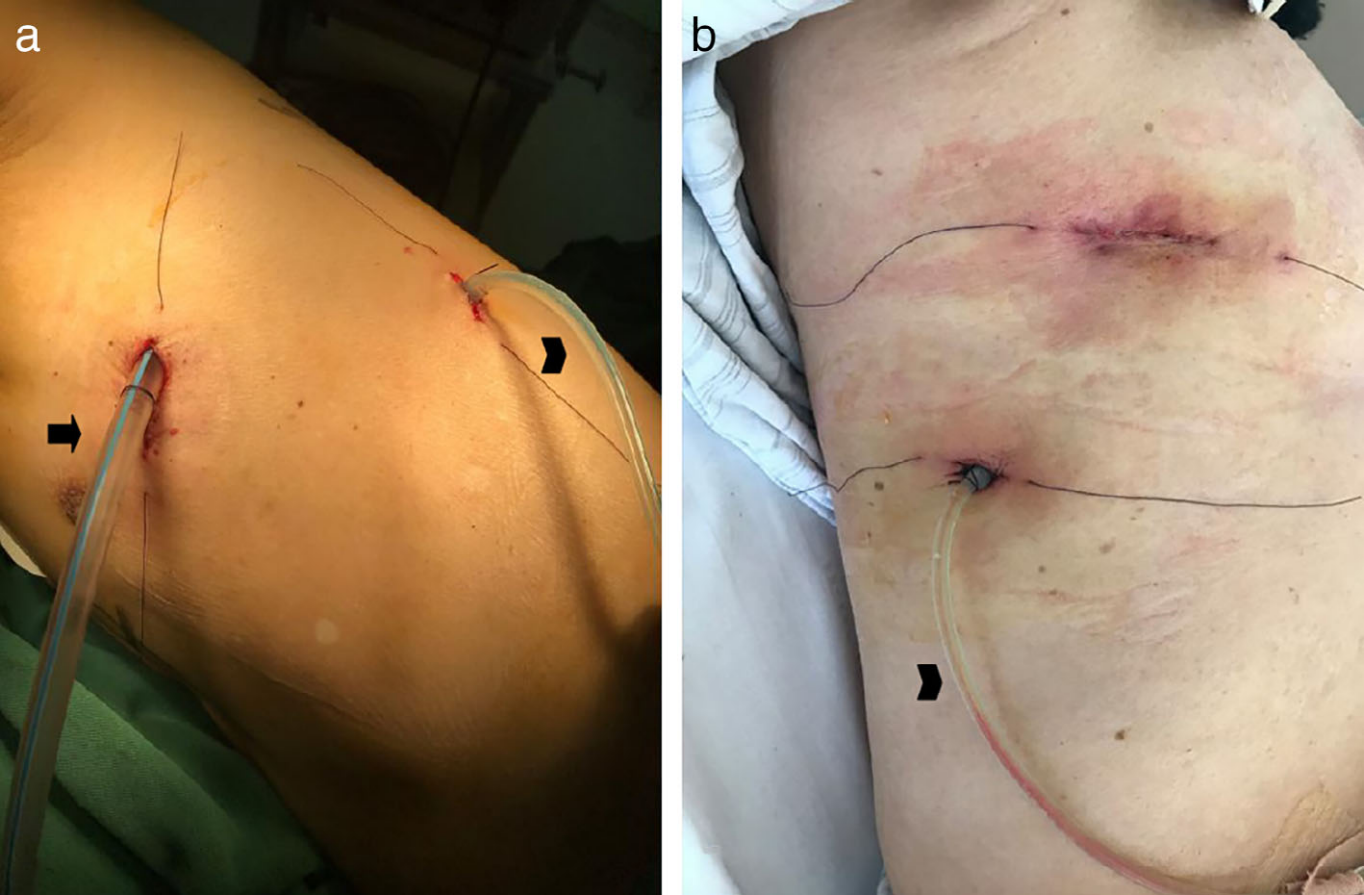
(**a**) Patient accepted left upper lobectomy in Group ball. One commonly used chest tube (28F, arrow) and one drainage ball (arrowhead) were inserted. (**b**) Patient accepted right lower lobectomy in Group ball. One drainage ball (arrowhead) was inserted.

All patients were divided randomly into two groups. Patients who were diagnosed with invasive carcinoma preliminarily and met all other criteria gave their informed consent before randomization. The Research Unit of the In Patient Department randomized the patients by using sequentially numbered, opaque, sealed envelopes. Patients were finally enrolled into the study by thoracic surgeons during surgery when they were diagnosed with invasive carcinoma definitively by frozen section. In the first group (Group tube), a commonly used chest tube (28F, Protex, Smiths Medical International Limited, Hythe, Kent, UK) was placed in the basal position without negative pressure suction at the end of the lobectomy procedure (Fig 1). In the second group (Group ball), the commonly used chest tube was replaced with a drainage ball (diameter 5 mm) which has the function of negative pressure suction and its actual value of negative pressure will decrease when the drainage ball is filled (Fig 1). These above mentioned tubes which were placed in the basal position at the seventh intercostal space in order to drain the fluid were named lower tube. In both groups, patients undergoing upper lobectomy had one upper tube (28F) inserted in the apical position to promote recruitment (Figs 2, 3). The operation was performed under general anesthesia with lung exclusion by double lumen intubation in both groups. Lobectomy and systemic lymph node dissection were performed by three surgeons (J.Z., C.L., and C.X.) using a two‐port anterior approach without rib spreading. A 2.5 cm incision was made at the fourth intercostal space along the anterior axillary line; another 1 cm incision was made at the seventh intercostal space alone the mid‐axillary line. A 10 mm 30 degree thoracoscope (Karl Storz, Tuttlingen, Germany) was placed at the 1 cm incision. Harmonic scalpel (Ethicon Endo‐Surgery Inc., Cincinnati, OH) and electrocautery were used for routine energy devices. The bronchus, vessel and fissure were managed with endoscopic staplers (Ethicon Endo‐Surgery Inc., Cincinnati, OH). Surgeons had previous experience of >300 lobectomies. According to the results of frozen section, all patients who were diagnosed as invasive carcinoma were accepted for systemic lymph node dissection. Before the incisions were sewn up, a submersion test was performed in order to detect the defects of lung and bronchial stump. These defects were sealed with nonabsorbable (Prolene 4‐0, Johnson & Johnson, Cincinnati, OH) suture. The incisions were then closed with absorbable (Vicryl 3–0, Johnson & Johnson, Cincinnati, OH) suture. Anesthetists then re‐expanded the remaining lobes before anesthesic recovery by lumen intubation. No basic postoperative analgesics were used.

The data collected include age, gender, lung function, drainage volume, drainage time, forced expiratory volume in one second (FEV1), length of stay, postoperative pain score according to the Visual Analogue Scale (VAS) within 24 hours after surgery and chest tube removal. Nurses recorded drainage volume every 24 hours and evacuated the drainage ball every two hours to keep it in the negative pressure state. In both groups, the earliest time allowed for removal was 24 hours after surgery, and for this it was required that total volume of <200 mL every 24 hours, fluid production was serous with no air leakage.

Chest X‐rays were routinely obtained on the first day after operation, the day of removal of the chest tube and again 10–14 days following surgery in the outpatient clinic. Postoperative pain was assessed and scored from 0 to 10 according to VAS by the surgeon. After surgery, pain assessment was carried out on the first day after the operation had taken place and the day of removal of the chest tube. The analgesic Tramadol 50 mg was administered to patients by intramuscular injection when pain affected their recovery. The amount of tramadol used was recorded. Patients were allowed to leave hospital when the last tube was removed and their blood and electrolyte tests were normal. The study endpoint was the last patient to leave the hospital.

All statistical analyses were performed using SPSS 20.0 statistical software. Numerical data are expressed as the mean ± SD. Variables were compared using the two‐sample *t*‐test. The significance level was set as *P* < 0.05.

## Results

From July 2018 to September 2018, a total of 60 patients were enrolled into the study and accepted the different drainage protocol. Patient characteristics are listed in Table 1 and there was no significant difference between the features of each group.

**Table 1:** Patient characteristics and operative factors

	Group tube (*n* = 30)	Group ball (*n* = 30)	*P*‐value
Gender			
Female	18	17	
Male	12	13	
Age (year)	55.53 ± 8.79	57.03 ± 9.74	0.534
FEV1/FVC (%)	73.43 ± 2.79	72.47 ± 2.75	0.182
FEV1 (%)	84.33 ± 9.17	81.03 ± 11.30	0.219
Lobes removed			
LUL	8	9	
LLL	2	5	
RUL	12	10	
RLL	8	6	
Histology			
Adenocarcinoma	16	17	
Squamous carcinoma	11	9	
Mucinous carcinoma	1	0	
Others	2	4	
Blood loss (mL)	47.33 ± 23.81	51.33 ± 23.15	0.512
Operation time (minutes)	148.47 ± 23.68	150.83 ± 23.14	0.697

LLL, left lower lobe; LUL, left upper lobe; RLL, right lower lobe; RUL, right upper lobe.

There was no significant difference between the two groups in terms of total drainage volume (1100.67 ± 661.62 vs. 1017.67 ± 373.76 mL, *P* = 0.552). Drainage time was less in patients in Group ball (upper tube: 3.89 ± 1.63 days vs. 5.10 ± 2.02 days, *P* = 0.048; lower tube: upper lobe 4.84 ± 1.61 days vs. 5.90 ± 1.52 days, *P* = 0.041, lower lobe 3.82 ± 1.08 days vs. 5.70 ± 2.63 days, *P* = 0.042). Following extubation there was no incidence of clinically significant subcutaneous emphysema, pleural effusion, or pneumothorax necessitating drain reinsertion in either group. Group tube patients had a significantly higher pain score (after operation: 6.20 ± 1.56 vs. 3.47 ± 1.80, *P* < 0.001; after removal of chest tube: 3.00 ± 1.29 vs. 1.47 ± 1.28, *P* < 0.001) and more analgesic was used (5.00 ± 3.24 times vs. 2.83 ± 2.09 times, *P* = 0.003) compared to Group ball patients. Meanwhile, Group ball patients had shorter length of stay compared to Group tube patients (5.93 ± 2.48 days vs. 8.30 ± 3.24 days, *P* = 0.002). Postoperative complications included constant air‐leakage (Group tube: *n* = 2), infection (Group tube: *n* = 1) and cerebral infarction (Group ball: *n* = 1). There was no significant difference between each group. The results are listed in Table 2.

**Table 2:** Results of study

	Group tube (*n* = 30)	Group ball (*n* = 30)	*P‐*value
Drainage (mL)	1100.67 ± 661.62	1017.67 ± 373.76	0.552
Extubation days			
Upper tube	5.10 ± 2.02	3.89 ± 1.63	0.048
Lower tube			
Upper lobe	5.90 ± 1.52	4.84 ± 1.61	0.041
Lower lobe	5.70 ± 2.63	3.82 ± 1.08	0.042
VAS score			
After operation	6.20 ± 1.56	3.47 ± 1.80	<0.001
After removal	3.00 ± 1.29	1.47 ± 1.28	<0.001
Analgesic (times)	5.00 ± 3.24	2.83 ± 2.09	0.003
Length of stay	6.37 ± 1.99	5.4 ± 1.65	0.045
Complications			
Air‐leakage	2	0	0.155
Infection	1	0	0.321
Cerebral infarction	0	1	0.321

The most important notable event in our study was air leaking around tubes. We conducted reinforced suture in four patients; three Group tube patients and one Group ball patient, and the leaks were sealed successfully. It is worth mentioning that this situation only happens with the commonly used chest tube.

## Discussion

A minimally invasive thoracic technique provides smaller incision, less postoperative pain, shorter recovery period and reduces risk of intervention after chest tube removal compared with thoracotomy.^12^ As a result of this, video‐assisted thoracoscopic surgery (VATS) lobectomy has become the gold standard treatment for early stage, non‐small cell lung cancer (NSCLC) according to NCCN guidelines. Even so, postoperative chest tube management is indispensable and the pain caused by tube insertion is significant.^13^ Pain control plays a significant role in post‐lobectomy recovery and complications. Effective pain management helps in early lung recruitment through deep breathing exercises, better cough up of phlegm, reduces the incidence of lung infections, and enables earlier out‐of‐bed mobilization. In our study, Group ball achieved less VAS scores and used fewer analgesics. In both groups, almost all the maximum pain scores were recorded within the first 24 hours after surgery and different chest tubes influenced the maximum pain scores. Factors may include: (i) A smaller tube does not impinge on the neurovascular bundle or alter the geometry of the intercostal space^14^ and (ii) a drainage ball is made of a more soft and flexible material so that makes patients feel more comfortable.^15^ The main limitation of our study was that the upper tube may have caused more pain and produced more fluid by stimulating the pleura, although no significant difference was observed in drainage of both groups.

We found a significant difference in postoperative hospitalization and extubation days between the groups. The correlation between these two outcomes may prove that chest tube management plays a key role in the ERAS of lobectomy and that our criteria of extubation were advisable. First, patients in Group ball coughed up phlegm effectively because of less pain enabling accelerated lung re‐expansion after surgery. Second, because of the portability of the drainage ball, patients were able to get out of bed and resume normal activities more quickly. These reasons contributed to a faster recovery and shorter hospital stay. However, for buffering and preventing the pneumothorax after removal, we did not remove the upper and lower tubes together within 24 hours for anyone accepted for upper lobectomy, although they met the criteria for removal. It led to hospitalization being prolonged and an increase in costs.

Drainage of pleural fluid is one of the main functions of a chest tube. On one hand, chest tubes can be obstructed by accumulation of clots, debris or other fluids and smaller drains have been found to be less effective and more prone to the risk of obstruction^16^ possibly causing serious clinical consequences. The study by Clark *et al*. demonstrates this.^17^ On the other hand, several retrospective studies highlight that small chest tubes can in some cases replace large chest tubes.^15^, ^18^, ^19^, ^20^ However, we have not experienced obstruction of a drainage ball in our study and the drainage volume of the two groups was similar. This beneficial result may be because of the negative pressure suction of the drainage ball but further studies are needed to quantify this.

Pleural suction is frequently exerted after lobectomy. Many studies have attempted to research the correlation of pleural suction and air leakage.^21^, ^22^ Some have concluded that pleural suction will not reduce the incidence rate of long‐time air leak. However, Varela *et al*. indicated postoperative pleural pressures are varied and concluded that for patients accepted for upper lobectomy, pleural suction can largely decrease the differential pleural pressure.^23^ Recently, Aguayo *et al*. proved that under the correct conditions, pleural suction may enhance recovery after thoracotomy.^24^ Using a drainage ball with a low negative pressure of 10 kpa for drainage is a promising approach to prevent potential dangers from high negative pressure and it has been proven may enhance recovery after lobectomy in our study.^25^ However, the specific mechanism needs to be demonstrated by more research. Moreover, the drainage ball system is cheaper than other suction devices thereby relieving the financial burden of patients and society.

With the development of ERAS in thoracic surgery, previous studies have reported on using a chest tube from various aspects such as caliber,^15^, ^18^, ^19^, ^20^, ^26^ material^14^ and early removal^27^, ^28^ and inspired us to conduct this study. However, extrapolation of our findings to other thoracic surgeons is limited because our data were collected from a single thoracic center and we hope that our subsequent trials will go further.

For patients undergoing lobectomy, a drainage ball with low negative pressure can reduce the length of hospitalization and postoperative pain compared with the more commonly used chest tube with water‐sealed bottle when a strict postoperative curative procedure is performed.

## Disclosure

The authors report no conflict of interest.
